# Serum levels and tissue expression of matrix metalloproteinase 2 (MMP-2) and tissue inhibitor of metalloproteinases 2 (TIMP-2) in colorectal cancer patients

**DOI:** 10.1007/s13277-013-1502-8

**Published:** 2014-01-07

**Authors:** Magdalena Groblewska, Barbara Mroczko, Mariusz Gryko, Anna Pryczynicz, Katarzyna Guzińska-Ustymowicz, Bogusław Kędra, Andrzej Kemona, Maciej Szmitkowski

**Affiliations:** 1 Department of Biochemical Diagnostics, University Hospital, Białystok, Poland; 2 Department of Biochemical Diagnostics, Medical University, Białystok, Poland; 3 Department of Neurodegeneration Diagnostics, Medical University, Białystok, Poland; 4 Second Department of General and Gastroenterological Surgery, Medical University, Białystok, Poland; 5 Department of General Pathomorphology, Medical University, Białystok, Poland

**Keywords:** Colorectal cancer, Matrix metalloproteinase 2, Tissue inhibitor of matrix metalloproteinases 2, Immunohistochemical staining

## Abstract

The objective of the study was the assessment of serum levels and tissue expression of matrix metalloproteinase 2 (MMP-2) and tissue inhibitor of matrix metalloproteinases 2 (TIMP-2) in patients with colorectal cancer (CRC). The study included 72 CRC patients and 68 healthy subjects. The serum levels of MMP-2 and TIMP-2 were measured using enzyme-linked immunosorbent assay (ELISA) method, whereas tissue expression of MMP-2 and TIMP-2 in cancer cells, interstitial inflammatory cells, and adjacent normal colorectal mucosa were examined by immunohistochemical staining of tumor samples. The serum levels of MMP-2 and TIMP-2 in cancer patients were significantly lower than those in control group, but the percentage of positive immunoreactivity of these proteins were higher in malignant and inflammatory cells as compared to normal tissue. There was a significant correlation between MMP-2 immunoreactivity in inflammatory cells and the presence of distant metastases and between TIMP-2 expression in inflammatory cells and tumor size, nodal involvement, and distant metastases. Area under receiver operating characteristic (ROC) curve (AUC) for serum MMP-2 was higher than for serum TIMP-2. Moreover, positive tissue expression of MMP-2 was a significant prognostic factor for CRC patients’ survival. Our findings suggest that MMP-2 and TIMP-2 might play a role in the process of colorectal cancer invasion and metastasis, but the significance of their interactions with tumor stroma and interstitial inflammatory infiltration in colorectal neoplasia require further elucidation.

## Introduction

Colorectal cancer (CRC) is one of the most frequent malignant tumors in the industrialized world [1]. Colorectal carcinogenesis is a complex, long-term process and includes several steps of malignant transformation from normal epithelium to cancer cells, which involves numerous genetic changes and results in various phenotypic alterations [2]. The next step of tumor development is the degradation of basement membrane and invasion of epithelium that leads to metastasis of cancer cells into distant organs [3]. Various proteases are involved in cancer progression and metastasis. Matrix metalloproteinases are synthesized by neoplastic and stromal cells [3]. In particular, matrix metalloproteinase 2 (MMP-2) and MMP-9, known as gelatinases, have been implicated to play a significant role in colon cancer progression and metastasis. Levels of the MMP-2 gene expression in colorectal tumors were lower than in adjacent normal mucosa and significantly correlated with the depth of invasion, venous invasion, and presence of liver metastasis [4]. Moreover, plasma MMP-2 levels were lower in CRC metastatic liver disease than in healthy controls [5]. However, Wu et al. revealed that the expression of MMP-2 protein was significantly increased in CRC tissues, whereas in normal colorectal cells, it was not detected [6].

Neoplastic tumors consist of cancer and nonmalignant cells, the latter also contribute in MMPs expression in tumors. These enzymes are expressed by fibroblasts, macrophages, monocytes, dendritic cells, natural killer cells, lymphocytes, and neutrophils [7]. MMP-2 mRNA in colon cancer tumors is mainly localized in fibroblasts, and it seems that the main source of MMP-2 in colorectal tumors is fibroblasts. However, a number of other cells involved in tumor biology have been found to express this proteinase. Thus, the expression of gelatinases in tumors is not only a consequence of their expression in cancer cells. Various immune cells express either MMP-2 or MMP-9 or both. The primary function of gelatinases is probably related with migration of immune cells. Cancer cells may use host cell gelatinases for their own purpose by modulation of MMP expression in host cells. Moreover, in combination with suppression of the immune response, they use host-derived gelatinases for their own advantage [8].

Remodeling of normal and tumor tissue may be a result of imbalance between MMPs, and their natural inhibitors—tissue inhibitors of matrix metalloproteinases (TIMPs) [9–12]. Li and coworkers [13] have shown that the imbalance between MMP-2 activity and its specific inhibitor TIMP-2 in colorectal tumor tissue might be a significant factor in the process of cancer invasion and metastasis. It was shown that the expression of MMP-2 in CRC tissues was significantly higher, but TIMP-2 was significantly lower than that in normal tissues [13]. Additionally, the MMP-2/TIMP-2 ratio was higher in CRC than in normal tissues and gradually decreased with tumor stage, depth of invasion, and lymph node metastasis [13]. In our previous study, the serum levels of MMP-2 and TIMP-2 were significantly lower in CRC patients than in healthy subjects and decreased with tumor stage [14]. However, according to the best of our knowledge, there is no study comparing serum levels of MMP-2 and TIMP-2 in colorectal cancer patients with their expression in colorectal tumor tissue: cancer cells, inflammatory infiltrate cells, and colorectal cells from adjacent normal tissue.

Therefore, the aim of the present study was to assess the concentrations of MMP-2 and TIMP-2 in the sera of CRC patients as well as immunohistochemical expression of these proteins in various types of cells in the tumors. We determined the serum levels and tumor tissue expression of MMP-2 and TIMP-2 in 72 CRC patients in relation to clinicopathological features of cancer as well as the prognostic significance of all variables tested for CRC patients’ survival. Moreover, we examined serum levels of MMP-2 and TIMP-2 in 68 healthy controls and assessed the areas under receiver operating characteristic (ROC) curves (AUCs) for both serum proteins.

## Material and methods

### Patients

The study included 72 previously untreated CRC patients which were diagnosed and operated on in the Second General and Gastroenterological Surgery Department of Białystok Medical University Hospital as well as a control group of 68 healthy volunteers. The study was approved by the local ethics committee, and the study number is R-I-002/443/2010. All the patients gave their informed consent to participate in the study.

In the clinical diagnosis of CRC patients, the physical examination, blood tests, chest X-rays, abdominal ultrasound, and computed tomography were used. A heart attack, heart failure, presence of extraintestinal tumors, and preoperative radio-chemotherapy were the factors which exclude patients from the study. The material obtained during colonoscopy and/or surgery from all cancer patients was subjected to microscopic evaluation.

Sixty-eight cancer patients underwent surgical tumor resection, whereas nonresectable tumors were diagnosed in four subjects. After routine histopathological analysis of tumor tissue, the cancer patients were assessed in accordance to Duke’s classification [15]: 1 patient in stage A, 4 patients in stage B1, 23 patients in stage B2, 19 patients in stage C1, 4 patients in stage C2, and 21 patients in stage D, and they were then subdivided into four groups depending on the infiltration of the bowel wall (T1, T2, T3, and T4), four groups in relation to nodal involvement (N0, N1, N2 and N3), and two groups regarding the absence or presence of distant metastases (M0 and M1). Eight colorectal cancer patients died of cancer during the 2-year observation period, whereas 42 patients survived. The number of patients in the analyzed subgroups is presented in Table 1.

**Table 1:** Characteristics of patients

Characteristics	No.
Colorectal cancer patients	72
Gender	
Female	29
Male	43
Age	
Median	69
Range	41–83
Tumor localization	
Colon	53
Rectum	19
Tumor stage (Duke’s classification)	
A	1
B1	4
B2	23
C1	19
C2	4
D	21
Depth of tumor invasion (T factor)	
T1	1
T2	4
T3	54
T4	13
Lymph node metastases (N factor)	
N0	30
N1	23
N2	9
N3	10
Distant metastases (M factor)	
M0	51
M1	21
Survival of patients (*n* = 50)	
Alive	42
Died of cancer	8
Resectability of tumor	
Resectable	68
Nonresectable	4

### Protein analyses

Venous blood samples from all the patients were drawn before any surgical treatment, chemo- or radiotherapy. All blood samples were allowed to clot before centrifugation. Sera were removed, aliquoted, and stored at −80 °C until assayed. Serum concentrations of MMP-2 and TIMP-2 were measured using enzyme-linked immunosorbent assay (ELISA) kits (R&D Systems, Abingdon, England) according to the manufacturer’s instructions. The serum samples were diluted 10-fold for the determination of MMP-2 and 50-fold for the measurement of TIMP-2. The intra-assay coefficient of variation (CV%) of MMP-2 is reported by the manufacturer to be 5.8 % at a mean concentration of 18.9 ng/mL, SD = 1.1, and that of TIMP-2 is reported to be 4.4 % at a mean concentration of 1.23 ng/mL, SD = 0.054. The reference cutoff values for serum levels of proteins tested were established previously [14]. The cutoff points were 160 ng/mL for MMP-2 and 85 ng/mL for TIMP-2. The positive results of MMP-2 and TIMP-2 are below cutoff values.

### Immunohistochemical staining

The tissue specimens were fixed in 10 % solution of buffered formalin and then dehydrated and embedded in paraffin. Obtained paraffin blocks were cut on microtome into 5-μm-thick sections, which were heated in a microwave to visualize antigen. For the detection of MMP-2 and TIMP-2 in the tissue samples, the mouse monoclonal antibodies (NCL-MMP2-507 and NCL-TIMP2-487, respectively) were used (Novocastra). The antigen–antibody complex was visualized by means of DAB chromogen (Novocastra). The immunoreactivity of MMP-2 and TIMP-2 in cancer cells was evaluated with light microscopy. Additionally, the intensity of the metalloproteinase and its inhibitor in inflammatory infiltrate cells (macrophages, polynuclears, and lymphocytes) in the neoplastic interstitium as well as in normal colorectal tissue from adjacent tumor mucosa was evaluated in the same tumor samples.

### Statistical analysis

The tumor stages A, B1, and B2 were analyzed as one group (stage A + B1 + B2) whereas stages C1 and C2 as stage C1 + C2 because of small numbers of patients in the particular subgroups. Similarly, the T1, T2, and T3 patients were analyzed as T1 + T2 + T3 subgroup whereas N1, N2, and N3 patients as N1 + N2 + N3 tumors.

The serum levels of MMP-2 and TIMP-2 did not follow a normal distribution based on Kolmogorov–Smirnov and Shapiro–Wilk tests. Therefore, nonparametric statistical analyses were used in the next step. The Mann–Whitney *U* test was used to compare the two groups in each category (CRC versus healthy controls; colon cancer versus rectal cancer; T1 + T2 + T3 versus T4 or N0 versus N1 + N2 + N3; M0 versus M1 group; resectable tumors versus nonresectable; the group of patients who survived versus patients who died of CRC). Differences between more than two groups (e.g., stages A + B1 + B2, C1 + C2, D) were compared using ANOVA on ranks (Kruskal–Wallis tests). If significant differences were found in Kruskal–Wallis test, we conducted the post hoc Dwass–Steele–Critchlow–Fligner test to determine which pairs of subgroups were different. Data are presented as median and range. Differences were considered statically significant with *p* values below 0.05.

Moreover, we calculated diagnostic criteria, such as ROC AUC for the MMP-2 and TIMP-2. The AUC for proteins tested was compared with AUC = 0.5 using the method described by Hanley and Hajian-Tilaki [16].

The tumor samples were indicated as positive and negative due to the presence or absence of MMP-2 or TIMP-2 immunoreactivity in three types of cells: cancer cells, interstitial inflammatory infiltrate, and normal colorectal tissue within tumor samples. The intensity of immune reaction of MMP-2 and TIMP-2 was evaluated in semiquantitative scale: 0 pt, no reaction; 1 pt, weak reaction; 2 pts, moderate reaction; and 3 pts, intense reaction. The immunoreactivity of each subgroup was analyzed as negative (0 pt) or positive (1 or 2 or 3 pts) expression. The presence of tissue expression of proteins tested is shown as a number of cases and percentages in each subgroup analyzed.

The correlations between tissue expression of MMP-2 and TIMP-2 and clinicopathological parameters of tumor were assessed using Fisher exact probability test and Fisher–Freeman–Halton test [17]. The Spearman rank correlation test was employed for the analyses of correlations between serum levels and tissue expression of MMP-2 and TIMP-2 in colorectal cancer patients. The patients’ univariate analyses were estimated using the log-rank test, and multivariate analyses employed Cox proportional hazards model. For all multivariate analyses, forward stepwise procedures were used. Statistical analyses were carried out using the STATISTICA 9.0 PL program (StatSoft, Inc., Tulsa, OK). Diagnostic criteria and the ROC curves were calculated using MedCalc statistical software (MedCalc Software, Mariakerke, Belgium) and Microsoft Office Excel program (Microsoft Corporation, Redmond, WA).

## Results

### Serum levels of MMP-2 and TIMP-2 in colorectal cancer patients in relation to clinicopathological features of tumor

Concentrations (median and range) of MMP-2 and TIMP-2 in the sera of colorectal cancer patients and healthy subjects are presented in Table 2. Serum levels of MMP-2 and TIMP-2 were significantly lower in CRC patients than in healthy controls. Serum levels of MMP-2 and TIMP-2 correlated with tumor stage and were the lowest in CRC patients with stage D. Concentrations of proteins tested were also lower in the sera of patients with T4 tumors than in T1 + T2 + T3 group and in CRC subjects with lymphatic nodes involved (N1 + N2 + N3 subgroup) than in patients without nodal metastases (N0). Moreover, in patients with distant metastases, the serum levels of MMP-2 and TIMP-2 were lower than those in M0 subgroup. All the differences did not reach a statistical significance.

**Table 2:** Serum levels of biomarkers tested in colorectal cancer patients in relation to clinicopathological features of tumor

Variable analyzed	No.	MMP-2 (ng/mL)		TIMP-2 (ng/mL)	
		Median (range)	*p*	Median (range)	*p*
Tested group					
CRC	72	180 (87–476)	<0.001*	81 (35–132)	0.003*
Control group	68	235 (108–384)		90 (54–141)	
Tumor stage (Duke’s classification)					
A + B1 + B2	28	180 (87–308)	0.548	82 (35–109)	0.804
C1 + C2	23	193 (110–361)		81 (68–109)	
D	21	170 (121–476)		79 (59–132)	
Tumor size (T factor)					
T1 + T2 + T3	59	182 (87–479)	0.067	82 (35–132)	0.707
T4	13	136 (110–361)		79 (59–110)	
Nodal involvement (N factor)					
N0	30	182 (87–308)	0.851	82 (35–109)	0.621
N1 + N2 + N3	42	175 (110–476)		80 (59–132)	
Distant metastases (M factor)					
M0	51	182 (87–361)	0.372	82 (35–109)	0.733
M1	21	170 (121–476)		79 (59–132)	

*p < 0.05, statistically significant

### The associations between the expression of proteins tested and clinicopathological features of tumor

**Fig. 1:**
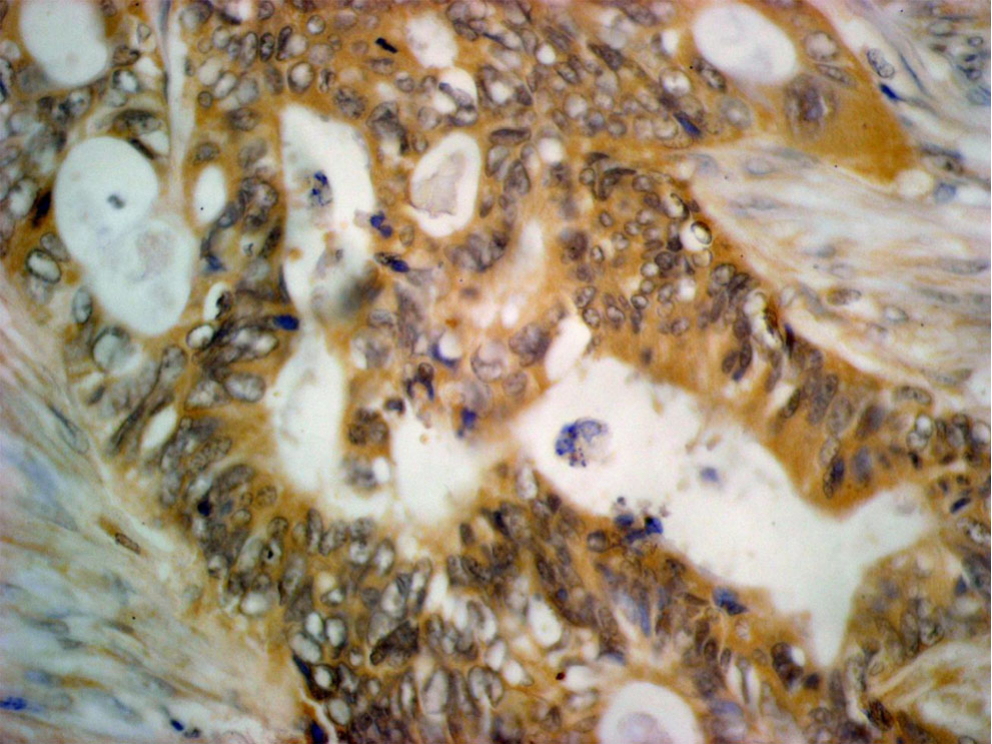
Immunohistochemical staining. MMP-2 expression in cancer cells (+2); original magnification, ×400

Table 3 presents the associations between the expression of MMP-2 and TIMP-2 in various types of cells and clinicopathological features of tumor. In colorectal cancer, the positive reaction was observed in 23.6 % of cases in cancer cells (Fig. 1) and 50 % of inflammatory infiltrate cells, whereas in normal colorectal tissue surrounding tumor nests, the presence of MMP-2 was observed only in one case.

**Table 3:** The relationship between clinicopathological features of tumor and the expression of MMP-2 and TIMP-2

Variable analyzed	MMP-2									TIMP-2								
	Colorectal cancer cells			Inflammatory infiltrate cells			Normal colorectal tissue			Colorectal cancer cells			Inflammatory infiltrate cells			Normal colorectal tissue		
	Negative	Positive	*p*	Negative	Positive	*p*	Negative	Positive	*p*	Negative	Positive	*p*	Negative	Positive	*p*	Negative	Positive	*p*
	*n* (%)	*n* (%)		*n* (%)	*n* (%)		*n* (%)	*n* (%)		*n* (%)	*n* (%)		*n* (%)	*n* (%)		*n* (%)	*n* (%)	
Colorectal cancer	55 (76.4)	17 (23.6)		36 (50.0)	36 (50.0)		71 (98.6)	1 (1.4)		9 (12.5)	63 (87.5)		18 (25.0)	54 (75.0)		61 (84.7)	11 (15.3)	
Tumor stage (Duke’s classification)																		
A + B1 + B2	19 (67.9)	9 (32.1)	0.181	11 (39.3)	17 (60.7)	0.070	28 (100.0)	0 (0.0)	0.292	4 (14.3)	24 (85.7)	0.820	6 (21.4)	22 (78.6)	0.076	26 (92.9)	2 (7.1)	0.083
C1 + C2	17 (73.9)	6 (26.1)		10 (43.5)	13 (56.5)		23 (100.0)	0 (0.0)		2 (8.7)	21 (91.3)		3 (13.0)	20 (87.0)		16 (69.6)	7 (30.4)	
D	19 (90.5)	2 (9.5)		15 (71.4)	6 (28.6)		20 (95.2)	1 (4.8)		3 (14.3)	18 (85.7)		9 (42.9)	12 (57.1)		19 (90.5)	2 (9.5)	
Tumor size (T factor)																		
T1 + T2	4 (80.0)	1 (20.0)	0.336	1 (20.0)	4 (80.0)	0.496	5 (100.0)	0 (0.0)	1.000	1 (20.0)	4 (80.0)	0.215	0 (0.0)	5 (100.0)	0.003*	4 (80.0)	1 (20.0)	0.863
T3	43 (79.6)	11 (20.4)		28 (51.9)	26 (48.1)		53 (98.1)	1 (1.9)		5 (9.3)	49 (90.7)		10 (18.5)	44 (81.5)		46 (85.2)	8 (14.8)	
T4	8 (61.5)	5 (38.5)		7 (53.8)	6 (46.2)		13 (100.0)	0 (0.0)		3 (23.1)	10 (76.9)		8 (61.5)	5 (38.5)		11 (84.6)	2 (15.4)	
Nodal involvement (N factor)																		
N0	20 (66.7)	10 (33.3)	0.155	12 (40.0)	18 (60.0)	0.112	30 (100.0)	0 (0.0)	0.125	4 (13.3)	26 (86.7)	0.181	6 (20.0)	24 (80.0)	0.007*	27 (90.0)	3 (10.0)	0.107
N1	17 (73.9)	6 (26.1)		10 (43.5)	13 (56.5)		23 (100.0)	0 (0.0)		1 (4.3)	22 (95.7)		2 (8.7)	21 (91.3)		16 (69.6)	7 (30.4)	
N2	9 (100.0)	0 (0.0)		6 (66.7)	3 (33.3)		8 (88.9)	1 (11.1)		3 (33.3)	6 (66.7)		4 (44.4)	5 (55.6)		9 (100.0)	0 (0.0)	
N3	9 (90.0)	1 (10.0)		8 (80.0)	2 (20.0)		10 (100.0)	0 (0.0)		1 (10.0)	9 (90.0)		6 (60.0)	4 (40.0)		9 (90.0)	1 (10.0)	
Distant metastases (M factor)																		
M0	36 (70.6)	15 (29.4)	0.125	21 (41.2)	30 (58.8)	0.037*	51 (100.0)	0 (0.0)	0.292	6 (11.8)	45 (88.2)	0.714	9 (17.6)	42 (82.4)	0.036*	42 (82.4)	9 (17.6)	0.491
M1	19 (90.5)	2 (9.5)		15 (71.4)	6 (28.6)		20 (95.2)	1 (4.8)		3 (14.3)	18 (85.7)		9 (42.9)	12 (57.1)		19 (90.5)	2 (9.5)	

*p < 0.005, statistically significant

The percentage of MMP-2-positive cases decreased with tumor stage; it was the lowest in stage D as well in cancer cells as in interstitial inflammatory infiltrate cells. Similar observations were made in inflammatory cells in relation to tumor size (T factor) and nodal involvement (N factor); the highest number of positive cases was seen in T1 + T2 subgroup and in N0 patients. Moreover, in patients without distant metastases (M0 subgroup), the percentages of positive MMP-2 staining were higher than those in M1 subgroup, although the differences between M0 and M1 tumors were significant only for inflammatory cells (*p* = 0.037).

**Fig. 2:**
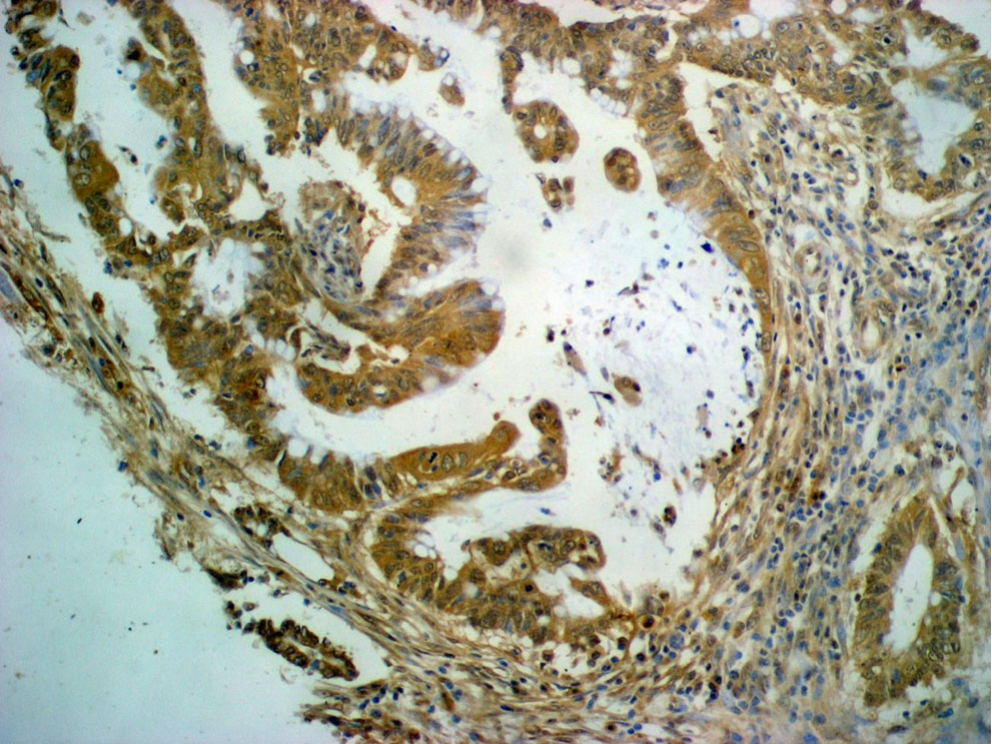
Immunohistochemical staining. TIMP-2 expression in cancer cells (+3); original magnification, ×400

The correlations between the expression of TIMP-2 and clinicopathological features of tumor are also presented in Table 3. The positive reaction was observed in 87.5 % of cancer cells (Fig. 2), 75 % of inflammatory infiltrate cells, and 15.3 % of normal cells. The percentages of positive cases were the highest in stage C1 + C2 in all types of cells, although the differences between tumor stages did not reach a statistical significance. The ratio of TIMP-2-positive cases varied in relation to T factor; the lowest number of positive cases was observed in T4 tumors, whereas the highest percentage of TIMP-2-positive cases among cancer cells was observed in T3 subgroup, but in T1 + T2 tumors—in 100 % of inflammatory cells (statistically significant difference with *p* = 0.003). Similarly, the number of TIMP-2-positive cases varied with the number of lymphatic nodes involved. It was both in cancer and inflammatory cells that the highest number of positive cases was observed in N1 tumors, although the differences within N subgroup were significant only for the expression of TIMP-2 in inflammatory cells (*p* = 0.007). The presence of TIMP-2 in inflammatory cells was also significantly higher (*p* = 0.036) in M0 patients than in M1 subgroup.

### Correlations between serum levels and tissue expression of MMP-2 and TIMP-2 in colorectal cancer patients

Table 4 presents the associations between serum levels of MMP-2 and TIMP-2 in CRC patients and the presence of the expression of these proteins in three types of cells: colorectal cancer cells, interstitial inflammatory infiltrate cells, and normal colorectal cells. Serum levels of MMP-2 were significantly higher in patients with positive expression of this protein in cancer cells (*p* = 0.047) as well as in patients with positive expression of TIMP-2 in inflammatory (*p* < 0.001) and normal colorectal cells (*p* = 0.04).

**Table 4:** Serum levels of biomarkers tested in colorectal cancer patients in relation to presence of MMP-2 and TIMP-2 expression in colorectal cancer cells, interstitial inflammatory infiltrate cells and in normal colorectal tissue

Variable analyzed	No.	Serum MMP-2 (ng/mL)		Serum TIMP-2 (ng/mL)	
		Median (range)	*p*	Median (range)	*p*
Expression of MMP-2 in colorectal cancer cells					
Negative	55	176 (87–476)	0.047*	81 (35–132)	0.162
Positive	17	202 (145–361)		90 (64–110)	
Expression of MMP-2 in inflammatory cells					
Negative	36	172 (87–296)	0.128	83 (35–110)	0.761
Positive	36	185 (115–476)		81 (36–132)	
Expression of MMP-2 in normal colorectal tissue					
Negative	71	179 (87–476)	0.548	82 (35–132)	0.194
Positive	1	204		69	
Expression of TIMP-2 in colorectal cancer cells					
Negative	9	175 (114–230)	0.319	85 (73–92)	0.615
Positive	63	182 (87–476)		81 (35–132)	
Expression of TIMP-2 in inflammatory cells					
Negative	18	150 (87–230)	<0.001*	78 (35–106)	0.080
Positive	54	199 (115–476)		83 (61–132)	
Expression of TIMP-2 in normal colorectal tissue					
Negative	61	175 (87–476)	0.040*	80 (35–132)	0.161
Positive	11	207 (159–3,170)		90 (68–110)	

*p < 0.05, statistically significant

These observations were confirmed in Spearman correlation analysis (Table 5). The serum levels of MMP-2 correlated significantly with serum levels of TIMP-2 in colorectal cancer patients (*p* < 0.001) and intensity of this protein expression in inflammatory (*p* = 0.012) and normal colorectal cells (*p* = 0.039). Moreover, the intensity of MMP-2 expression in inflammatory cells correlated significantly with the expression of this protein in cancer cells (*p* = 0.006) and with the expression of TIMP-2 in all types of cells analyzed (*p* = 0.040, *p* = 0.003, and *p* = 0.0495, respectively). The expression of TIMP-2 in inflammatory cells correlated significantly with the expression of this protein in cancer and normal cells (*p* < 0.001 and *p* = 0.004, respectively).

**Table 5:** Correlations between serum levels and tissue expression of MMP-2 and TIMP-2 in colorectal cancer patients

Variable analyzed	Serum TIMP-2	Tissue MMP-2 in cancer cells	Tissue MMP-2 in inflammatory cells	Tissue MMP-2 in normal cells	Tissue TIMP-2 in cancer cells	Tissue TIMP-2 in inflammatory cells	Tissue TIMP-2 in normal cells
	*r* (*p*)	*r* (*p*)	*r* (*p*)	*r* (*p*)	*r* (*p*)	*r* (*p*)	*r* (*p*)
Serum MMP-2	0.51 (<0.001*)	0.22 (0.064)	0.14 (0.239)	0.07 (0.551)	0.09 (0.458)	0.29 (0.012*)	0.24 (0.039*)
Serum TIMP-2		0.16 (0.187)	0.02 (0.844)	−0.15 (0.196)	−0.03 (0.802)	0.17 (0.153)	0.17 (0.163)
Tissue MMP-2 in cancer cells			0.32 (0.006*)	−0.07 (0.585)	0.06 (0.625)	0.11 (0.347)	0.12 (0.304)
Tissue MMP-2 in inflammatory cells				0.09 (0.442)	0.24 (0.040*)	0.35 (0.003*)	0.23 (0.050*)
Tissue MMP-2 in normal cells					−0.11 (0.365)	−0.02 (0.899)	−0.05 (0.674)
Tissue TIMP-2 in cancer cells						0.47 (<0.001*)	0.13 (0.264)
Tissue TIMP-2 in inflammatory cells							0.34 (0.004*)

*r* indicates correlation coefficient

*p < 0.005, statistically significant

### Serum and tissue expression of MMP-2 and TIMP-2 as prognostic factors of patients’ survival

Univariate regression analysis showed that tumor stage (*p* = 0.027), presence of nodal (*p* = 0.018) and distant metastases (*p* = 0.006), tumor resectability (*p* = 0.002), and presence of MMP-2 expression in normal colorectal cells (*p* = 0.030) were significant factors for patients’ 2-year survival (Table 6). Neither age, gender of CRC patients, and T factor nor serum levels and expression of both proteins tested in other types of cells were significant prognostic factors. Multivariate regression analysis with Cox proportional hazard model revealed that none of factors analyzed were an independent prognostic factor for the survival of CRC patients.

**Table 6:** Univariate Cox analysis of regression

Variable tested	*p*	HR
Age	0.997	1.000
Tumor stage (Duke’s classification)	0.086	
C1 + C2 vs A + B1 + B2	0.970	0.000
D vs A + B1 + B2	0.027*	11.259
Tumor size (T factor)	0.230	
T3 vs T2	0.962	9,271.216
T4 vs T2	0.956	34,627.135
Nodal involvement (N factor)	0.127	
N1 vs N0	0.967	0.000
N2 vs N0	0.018*	14.347
N3 vs N0	0.054	11.127
Distant metastases (M factor)	0.006*	20.674
Resectability of tumor	0.002*	0.073
Serum MMP-2	0.968	1.000
Serum TIMP-2	0.662	1.009
Tissue MMP-2 in cancer cells	0.498	0.480
Tissue MMP-2 in inflammatory cells	0.331	2.281
Tissue MMP-2 in normal cells	0.030*	11.361
Tissue TIMP-2 in cancer cells	0.551	24.715
Tissue TIMP-2 in inflammatory cells	0.141	0.299
Tissue TIMP-2 in normal cells	0.445	0.035

*p < 0.005, statistically significant

### Diagnostic criteria for MMP-2 and TIMP-2 in CRC patients

**Fig. 3:**
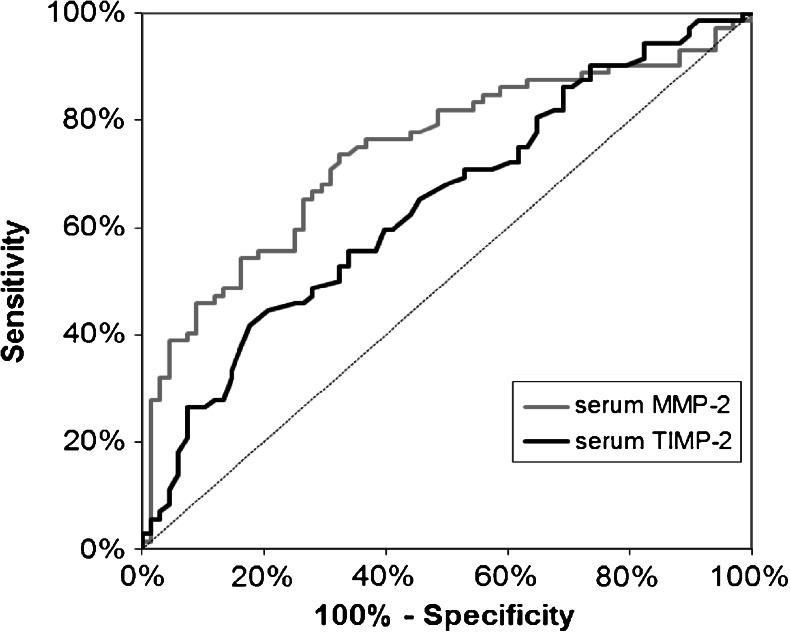
ROC curves for serum levels of biomarkers analyzed: MMP-2 (AUC = 0.7402) and TIMP-2 (AUC = 0.6432)

The ROC AUC for MMP-2 (0.7402) was higher than the AUC for TIMP-2 (0.6432) (Fig. 3). Additionally, AUC for both proteins was significantly higher than value AUC = 0.5 (*p* < 0.001 for MMP-2 and *p* = 0.002 for TIMP-2).

## Discussion

Expression of genes encoding type IV collagen-degrading metalloproteinases and tissue inhibitors of metalloproteinases was detected in various human tumor cells, including colorectal cancer [18]. Transcripts for MMP-2 were more frequently expressed in mesenchymal tumor cells than in epithelial tumor cells. Their activity was regulated by TIMPs, which are induced mainly in stromal cells [18]. The immunohistochemical expression of MMP-2 in CRC tumors is significantly higher than that in adjacent normal tissues [13, 19]. However, little is known about the comparison of the levels of MMP-2 and TIMP-2 in the sera of CRC patients with an expression of MMP-2 and its inhibitor in colorectal cancer tissue. Therefore, the aim of the study was to examine the serum levels of MMP-2 and TIMP-2 in CRC patients and to compare the serum concentrations of those proteins with their presence and intensity expression in colorectal cancer cells, interstitial infiltrate inflammatory cell, and normal colorectal cells. We have also assessed the significance of MMP-2 and TIMP-2 as prognostic factors of CRC patients’ survival.

In our study, the serum levels of MMP-2 and TIMP-2 in CRC patients and healthy controls were assessed using an ELISA method. We revealed that the concentrations of both proteins tested were significantly lower in cancer patients than in healthy subjects, which is in agreement with our previous results [14] as well as with the investigation of Waas et al., who have shown that plasma pro-MMP-2 levels were lower in colorectal cancer patients than in healthy controls [20]. Moreover, in the study of Oberg et al., the serum levels of the MMP-2/TIMP-2 complexes were significantly lower in CRC patients as compared to healthy blood donors; however, the serum levels of free MMP-2 and total TIMP-2 were significantly higher in comparison with control group [21].

In the present paper, the concentrations of MMP-2 and TIMP-2 in the sera of CRC patients revealed neither any significant correlation with tumor stage, tumor size, and nodal involvement nor with the presence of distant metastases, although there was a tendency to decrease the levels of proteins tested in more advanced tumors. This tendency is opposite to the results obtained by of Angenete et al. who revealed that plasma levels of MMP-2 were higher in CRC patients with distant metastases [22]. However, our findings are in accordance with the study of Langenskiöld et al. who had found that plasma MMP-2 in CRC patients with T4 tumors was lower than that in T3 and T1 + T2 subgroups [23]. The decrease of serum MMP-2 and TIMP-2 in more advanced tumor stages might be caused by the formation of MMP–TIMP complexes in colorectal tumor progression. The differences between the results observed by cited authors and those obtained in our present study might be the effects of the complex function of MMP-2 and TIMP-2. It was suggested that MMP-2 inhibition occurs at high levels of TIMP-2, whereas low concentrations of the inhibitor are associated with the activation of MMP-2 [24, 25].

It was suggested that in cancer tissue, the enhanced expression of MMPs and TIMPs on the surface of inflammatory cells might be a result of a host response induced by tumors [26]. In the present paper, the tissue expression of MMP-2 and TIMP-2 was observed in cancer and interstitial inflammatory infiltrate cells as well as in normal colorectal epithelium. The expression of both proteins was higher in cancer cell than in normal tissue, where 15 % of normal colorectal cells expressed the presence of TIMP-2 and only 1 % of normal cells were MMP-2 positive, which is in line with the study of Gershtein et al., who revealed that the content of MMP-2 in tumors of patients with colorectal cancer was significantly increased in comparison with the adjacent normal mucosa [19]. Our results are also in accordance with the study of Wu et al., who demonstrated an increased expression of MMP-2 in CRC tissues, but negative in normal colorectal tissues [6], and with the paper of Kim et al. that also indicated higher levels of MMP-2 protein in colon and rectal tumor tissues than in corresponding paired normal mucosa, although, in their study, TIMP-2 tissue levels were significantly lower in cancer than in normal tissue [27]. Similarly, opposite results were obtained by Li et al., who revealed that TIMP-2 expression in CRC tissues was significantly lower than that in normal tissues [13]. Moreover, in our study, the highest percentage of positive reactions of MMP-2 was observed in interstitial inflammatory infiltrate cells. Obtained results may suggest that the main source of MMP-2 in cancer tissue is rather interstitial inflammatory cells from tumor microenvironment than malignant tumor cells. Our results may also confirm the role of inflammation in carcinogenesis of colorectal cells, which was suggested by other authors and in our previous study [28–30].

Interestingly, in both types of cells, the higher percentages of positive immunoreactivity were shown for TIMP-2 than for MMP-2; in cancer cells, we observed TIMP-2 immunostaining in 87.5 % cases in comparison with 23.6 % of MMP-2 positivity, whereas 75 % of inflammatory cells were TIMP-2 positive in comparison with 50 % of inflammatory cells with MMP-2 immunoreactivity. Our observations are in line with the results of Schwandner et al., who demonstrated that MMP-2 tumor expression was positive in 35 % of epithelial malignant cells, whereas there was an expression of TIMP-2 in 47 % of cases in rectal carcinoma [31].

In our current study, the correlations between expression of both proteins tested and clinicopathological features of CRC were evaluated. We did not confirm any significant correlation between MMP-2 and TIMP-2 expression in colorectal cancer cells or normal cells and tumor stage, tumor size (T), nodal involvement (N), presence of distant metastases (M), or resectability of tumor. In inflammatory cells, the expression of MMP-2 revealed a significant correlation with M factor and was higher in patients without distant metastases. The significant differences were also found for TIMP-2 expression in inflammatory infiltrate cell; the percentage of positive reactions decreased in patients with higher tumor size, number of lymph node involved, and metastases to distant organs. The opposite results were obtained by Jung et al., who demonstrated that the upregulation of TIMP-2 in submucosal colorectal cancer tissue was positively correlated with adjacent lymphatic vessel invasion and lymph node metastasis in submucosally invasive colorectal carcinoma [32].

Additionally, we compared serum levels of proteins tested with their tissue expression. We demonstrated that MMP-2 serum levels were significantly higher in patients with positive expression of this enzyme in cancer cells and in CRC patients with positive expression of TIMP-2 in inflammatory and normal cells. The observations are in agreement with the results of Spearman correlation test, which shows a significant positive association between serum MMP-2 concentration and expression of its inhibitor in the same types of cells as well as between serum levels of both proteins. These findings suggest a complex role of MMP-2/TIMP-2 network in colorectal cancer development and metastasis.

Strong expression of many MMPs has been related to poor survival of CRC patients. The expression of various TIMPs has been associated with both a beneficial and a poor outcome. Thus, there is a need to further clarify the significance of MMPs and TIMPs in CRC. In the present study, we also investigated whether the serum concentrations of MMP-2 and TIMP-2 and intensity of their expression in various types of cell might be useful prognostic factors for survival of CRC patients. The univariate regression analysis indicated only the presence of MMP-2 expression in normal colorectal cells as a significant prognostic factor for patients’ survival, although not independent on tumor stage. Our observations indicate that the expression of MMP-2 in normal mucosa of CRC patients could also be relevant as well to the outcome in cancer cells. Our results are also in line with the study of Langers et al. [33], who have shown that a high expression of MMP-2 in the normal colorectal mucosa was associated with reduced survival of CRC patients.

Additionally, our results show the tumor stage, nodal involvement, presence of distant metastases, and tumor resectability, but not serum MMP-2 nor TIMP-2 as the significant prognostic factors in CRC patients in univariate analysis. Obtained results are in agreement with our previous study [14], where preoperative serum MMP-2 and TIMP-2 levels were not prognostic markers for CRC patients. Similar observations were also demonstrated by Waas et al. [5], who showed that preoperative plasma pro-MMP-2 levels had no potential value as prognostic markers in colorectal cancer.

In the present paper, we defined the ROC AUC for the proteins tested to assess a potential clinical significance of MMP-2 and TIMP-2 in the diagnosis of CRC. We found that AUC for serum MMP-2 (0.740) was higher than for TIMP-2 (0.643), and both were significantly higher than AUC = 0.5. These results suggest the potential clinical usefulness of pretreatment serum MMP-2 and TIMP-2 as tumor markers in CRC.

In conclusion, the aim of the present study was to assess the serum levels of MMP-2 and TIMP-2 as well as tumor tissue expression of these proteins in patients with colorectal cancer. We have found the significant associations between decreased MMP-2 expressions in inflammatory cells and distant metastases as well as between tumor invasion and reduced expression of TIMP-2 in inflammatory interstitium. Moreover, the immunoreactivity of MMP-2 and TIMP-2 in colorectal cancer tissue correlated with the expression of these proteins in the interstitial inflammatory infiltrate cells as well as with the serum levels of TIMP-2 in CRC patients. The area under ROC curve for serum levels of proteins tested was higher for MMP-2 than for TIMP-2, what indicates a possible clinical significance of serum MMP-2 in the diagnosis of CRC, while the tissue expression of MMP-2 as a prognostic factor of patients’ survival. Our results suggest a complex network of interactions between tumor, its microenvironment, and stromal cells, but this issue requires further investigations.

## References

[1] Jemal A, Siegel R, Ward E, Hao Y, Xu J, Thun MJ (2009). Cancer statistics, 2009. CA Cancer J Clin.

[2] Fearon ER, Vogelstein B (1990). A genetic model for colorectal tumorigenesis. Cell.

[3] Vihinen P, Kahari VM (2002). Matrix metalloproteinases in cancer: prognostic markers and therapeutic targets. Int J Cancer.

[4] Oshima T, Kunisaki C, Yoshihara K (2008). Clinicopathological significance of the gene expression of matrix metalloproteinases and reversion-inducing cysteine-rich protein with Kazal motifs in patients with colorectal cancer: MMP-2 gene expression is a useful predictor of liver metastasis from colorectal cancer. Oncol Rep.

[5] Waas ET, Wobbes T, Ruers T, Lomme RM, Hendriks T (2006). Circulating gelatinases and tissue inhibitor of metalloproteinase-1 in colorectal cancer metastatic liver disease. Eur J Surg Oncol.

[6] Wu W, He JT, Ruan JD, Wang RB, Zhang YD (2008). Expression of MMP-2, MMP-9 and collagen type IV and their relationship in colorectal carcinomas. Xi Bao Yu Fen Zi Mian Yi Xue Za Zhi..

[7] Opdenakker G, Van den Steen PE, Van Damme J (2001). Gelatinase B: a tuner and amplifier of immune functions. Trends Immunol.

[8] Mook OR, Frederiks WM, Van Noorden CJ (2004). The role of gelatinases in colorectal cancer progression and metastasis. Biochim Biophys Acta.

[9] Talvensaari-Mattila A, Paakko P, Turpeenniemi-Hujanen T (2003). Matrix metalloproteinase-2 (MMP-2) is associated with survival in breast carcinoma. Br J Cancer.

[10] Kubben FJ, Sier CF, Meijer MJ (2006). Clinical impact of MMP and TIMP gene polymorphisms in gastric cancer. Br J Cancer.

[11] Giannopoulos G, Pavlakis K, Parasi A (2008). The expression of matrix metalloproteinases-2 and -9 and their tissue inhibitor 2 in pancreatic ductal and ampullary carcinoma and their relation to angiogenesis and clinicopathological parameters. Anticancer Res.

[12] Murnane MJ, Cai J, Shuja S, McAneny D, Klepeis V, Willett JB (2009). Active MMP-2 effectively identifies the presence of colorectal cancer. Int J Cancer.

[13] Li BH, Zhao P, Liu SZ, Yu YM, Han M, Wen JK (2005). Matrix metalloproteinase-2 and tissue inhibitor of metallo-proteinase-2 in colorectal carcinoma invasion and metastasis. World J Gastroenterol.

[14] Groblewska M, Mroczko B, Gryko M, Kędra B, Szmitkowski M (2010). Matrix metalloproteinase 2 and tissue inhibitor of matrix metalloproteinases 2 in the diagnosis of colorectal adenoma and cancer patients. Folia Histochem Cytobiol.

[15] Jass JR, Sobin LH, WHO International Histological Classification of Tumors (1989). Histological typing of intestinal tumors.

[16] Hanley JA, Hajian-Tilaki KO (1997). Sampling variability of nonparametric estimates of the areas under receiver operating characteristic curves: an update. Acad Radiol.

[17] Mehta CR, Patel NR (1986). Algorithm 643. FEXACT: a FORTRAN subroutine for Fisher’s exact test on unordered r×c contingency tables. TOMS.

[18] Sato H, Kida Y, Mai M (1992). Expression of genes encoding type IV collagen-degrading metalloproteinases and tissue inhibitors of metalloproteinases in various human tumor cells. Oncogene.

[19] Gershtein ES, Korotkova EA, Prorokov VV, Kushlinsky NE (2008). Matrix metalloproteinases 2, 3, 13 and their type 2 tissue inhibitor in tumors and plasma of patients with colorectal cancer. Bull Exp Biol Med.

[20] Waas ET, Hendriks T, Lomme RM, Wobbes T (2005). Plasma levels of matrix metalloproteinase-2 and tissue inhibitor of metalloproteinase-1 correlate with disease stage and survival in colorectal cancer patients. Dis Colon Rectum.

[21] Oberg A, Höyhtyä M, Tavelin B, Stenling R, Lindmark G (2000). Limited value of preoperative serum analyses of matrix metalloproteinases (MMP-2, MMP-9) and tissue inhibitors of matrix metalloproteinases (TIMP-1, TIMP-2) in colorectal cancer. Anticancer Res.

[22] Angenete E, Langenskiöld M, Falk P, Ivarsson ML (2007). Matrix metalloproteinases in rectal mucosa, tumour and plasma: response after preoperative irradiation. Int J Colorectal Dis..

[23] Langenskiöld M, Holmdahl L, Falk P, Ivarsson ML (2005). Increased plasma MMP-2 protein expression in lymph node-positive patients with colorectal cancer. Int J Colorectal Dis..

[24] Kinoshita T, Sato H, Okada A (1998). TIMP-2 promotes activation of progelatinase A by membrane-type 1 matrix metalloproteinase immobilized on agarose beads. J Biol Chem.

[25] Kurschat P, Zigrino P, Nischt R (1999). Tissue inhibitor of matrix metalloproteinase-2 regulates matrix metalloproteinase-2 activation by modulation of membrane-type 1 matrix metalloproteinase activity in high and low invasive melanoma cell lines. J Biol Chem.

[26] Liabakk NB, Talbot I, Smith RA, Wilkinson K, Balkwill F (1996). Matrix metalloprotease 2 (MMP-2) and matrix metalloprotease 9 (MMP-9) type IV collagenases in colorectal cancer. Cancer Res.

[27] Kim TD, Song KS, Li G (2006). Activity and expression of urokinase-type plasminogen activator and matrix metalloproteinases in human colorectal cancer. BMC Cancer.

[28] Kaminska JA, Kowalska MM, Nowacki MP, Chwalinski MG, Rysinska A, Fuksiewicz M (2000). CRP, TNFα, IL-1ra, IL-6, IL-8 and IL-10 in blood serum of colorectal cancer patients. Pathol Oncol Res.

[29] Kaminska J, Nowacki MP, Kowalska M (2005). Clinical significance of serum cytokine measurements in untreated colorectal cancer patients: soluble tumor necrosis factor receptor type I—an independent prognostic factor. Tumour Biol.

[30] Groblewska M, Mroczko B, Wereszczyńska-Siemiątkowska U (2008). Serum interleukin 6 (IL-6) and C-reactive protein (CRP) levels in colorectal adenoma and cancer patients. Clin Chem Lab Med.

[31] Schwandner O, Schlamp A, Broll R, Bruch HP (2007). Clinicopathologic and prognostic significance of matrix metalloproteinases in rectal cancer. Int J Colorectal Dis..

[32] Jung SA, Yang SK, Kim JS (2005). The expression of matrix metalloproteinases (MMPs), tissue inhibitor of metalloproteinases (TIMPs) and angiogenesis in relation to the depth of tumor invasion and lymph node metastasis in submucosally invasive colorectal carcinoma. Korean J Gastroenterol.

[33] Langers AM, Verspaget HW, Hawinkels LJ (2012). MMP-2 and MMP-9 in normal mucosa are independently associated with outcome of colorectal cancer patients. Br J Cancer.

